# Antiphospholipid Syndrome: A Comprehensive Clinical Review

**DOI:** 10.3390/jcm14030733

**Published:** 2025-01-23

**Authors:** Vasileios Patriarcheas, Georgios Tsamos, Dimitra Vasdeki, Elias Kotteas, Anastasios Kollias, Dimitris Nikas, Georgia Kaiafa, Evangelos Dimakakos

**Affiliations:** 1 First Propaedeutic Department of Internal Medicine, Aristotle University of Thessaloniki, AHEPA University Hospital, Stilponos Kyriakides 1 Str., 54636 Thessaloniki, Greece; vpatriar@gmail.com (V.P.); gdkaiafa@yahoo.gr (G.K.); 2 Second Propedeutic Department of Internal Medicine, Hippokration General Hospital, Aristotle University of Thessaloniki, Konstantinoupoleos 49 Str., 54942 Thessaloniki, Greece; tsamgeor@gmail.com; 3 Division of Endocrinology and Metabolism and Diabetes Centre, First Department of Internal Medicine, Medical School, Aristotle University of Thessaloniki, AHEPA University Hospital, Stilponos Kyriakides 1 Str., 54636 Thessaloniki, Greece; demivs14@gmail.com; 4 Oncology Unit, Third Department of Internal Medicine, Sotiria General Hospital for Chest Diseases, National and Kapodistrian University of Athens, 152 Mesogeion Ave., 11527 Athens, Greece; ilkotteas@med.uoa.gr; 5 Hypertension Center STRIDE-7, Third Department of Medicine, Sotiria Hospital, School of Medicine, National and Kapodistrian University of Athens, 152 Mesogeion Ave., 11527 Athens, Greece; taskollias@gmail.com; 6 Anatomy and Histology Laboratory, Nursing School, University of Athens, Papadiamantopoulou 123 Str., 11527 Athens, Greece; dimnikas@hotmail.com

**Keywords:** antiphospholipid syndrome, catastrophic antiphospholipid syndrome, lupus anticoagulant, anticardiolipin, anti β2-GPI, autoantibodies, thrombosis

## Abstract

**Background**: Antiphospholipid syndrome (APS) is a rare systemic autoimmune disease characterized by persistent antiphospholipid antibodies (aPL) in combination with recurrent thrombosis in the veins and/or arteries, obstetric morbidity, and various non-thrombotic associated complications. APS can be primary, as an isolated condition, or secondary in the context of another autoimmune disease, especially systemic lupus erythematosus. This comprehensive clinical review aims to summarize the current understanding of APS pathogenesis, diagnostic approaches, and treatment strategies for this unique clinical entity. **Methods**: A comprehensive review of the existing literature on APS was conducted, focusing on pathophysiological mechanisms, current diagnostic criteria, and therapeutic approaches. **Results**: APS pathogenesis involves complex interactions between aPL, phospholipid-binding proteins, and the coagulation cascade. Apart from the cardinal features of thrombosis and APS-related obstetric morbidity, APS is associated with a wide spectrum of clinical manifestations. Diagnosis remains challenging due to overlapping symptoms with other conditions, and clinicians should maintain a high index of suspicion in order to set the diagnosis. The recently published 2023 ACR/EULAR criteria although not definitive for clinical decision-making, these criteria offer clinicians a valuable tool to aid in determining whether further investigation for APS is warranted. Continued refinement of these criteria through ongoing feedback and updates is anticipated. Treatment strategies center on anticoagulation, but individualized approaches are necessary. **Conclusions**: Early diagnosis and multidisciplinary management of APS are critical to reducing morbidity and improving outcomes. Moreover, familiarization with the 2023 ACR/EULAR criteria is encouraged, recognizing that ongoing feedback and updates will contribute to their ongoing refinement and improvement. While VKAs remain the mainstay of treatment for most APS patients further research is needed to optimize treatment strategies and deepen our understanding of APS’s underlying disease mechanisms.

## 1. Introduction

Several discoveries and observations during the 20th century ultimately led to the identification of APS. It was back in 1906 when Wasserman developed the first serological assay for the diagnosis of syphilis. This test was dependent on a reaction between syphilis non-specific antibodies, known as reagins, with a reaction antigen, which later was identified as cardiolipin, an important component of the inner mitochondrial membrane. [1,2]. In the 1930s, it was estimated that syphilis affected more than 10 percent of the American population. In an effort to control this public health burden, the U.S. Congress passed the National Venereal Disease Control Act in 1938, which established measures for syphilis control and led to the wide use of Wasserman’s test by healthcare practitioners [3]. With the use of the test on large populations such as soldiers during the Second World War, it became clear that not all subjects with positive results were affected by syphilis, since they did not have clinical evidence of the disease, the so-called biologically false positive for syphilis serological test (BFP-STS). Over the next two decades, persistent BFP-STS was reported in systemic lupus erythematosus (SLE) patients. In 1952, Conley and Hartmann described two cases of SLE patients who had hemorrhages due to the presence of a circulating anticoagulant [3], which led to prolonged prothrombin–thrombin conversion, and five years later, it was discovered that the presence of this anticoagulant was related to BFP-STS [2]. Finally, in the 1970s, this anticoagulant action was shown to be caused by an acquired immunoglobulin (IgG or IgM) that was partially suppressed by the addition of phospholipids or platelets and was named “lupus anticoagulant” (LA), because it was initially thought to cause bleeding in SLE patients [2]. However, the term LA was a double misnomer, as it was not only detected in SLE patients (almost 50 percent of patients did not have SLE), and notwithstanding the fact that the antibody acts as an anticoagulant in vitro; in vivo, it was mainly associated with thrombotic events and less frequently with hemorrhage. In the same period, several cases connected the presence of LA with obstetric morbidity, and in the upcoming years there was clear evidence that LA is associated with recurrent miscarriage and intrauterine fetal death. The introduction of the anticardiolipin solid-phase immunoassay from the Graham Hughes group in London marked a significant step forward in the comprehension of APS [4]. The direct detection of anticardiolipin antibodies (aCL) led to the observation that there was a strong correlation between the IgG isotype of aCL antibodies and clinical thrombosis. Moreover, further studies demonstrated a relationship between aCL antibodies and the presence of LA [3,4].

All the above discoveries led to the description of a new syndrome by G.R.V. Hughes in his publication in the *British Medical Journal*, entitled “Thrombosis, abortion, cerebral disease, and the lupus anticoagulant” in 1983 [5]. Hughes was the first to originally characterize the interdisciplinary elements of the disease associated with the presence of not only LA but also aCL antibodies. Three years later, Hughes and colleagues observed that certain individuals who did not have SLE still developed the syndrome. The patients were categorized as having a primary APS according to Asherson’s classification of 1988 [6]. In the early 1990s, three different research groups made a significant breakthrough by simultaneously discovering a plasma protein cofactor required by aCL antibodies to bind to cardiolipin, which later was identified as Beta-2 Glycoprotein 1. Despite the fact that APS was described for the first time over 41 years ago, our understanding of this entity is continually evolving. APS constitutes a systemic autoimmune disease characterized by thrombotic events, obstetric morbidity (obstetric APS), and/or non-thrombotic complications, associated with the presence of persistently positive circulating antiphospholipid antibodies (aPL antibodies): anticardiolipin antibodies, anti-β_2_-glycoprotein 1 antibodies, and lupus anticoagulant. Anticardiolipin antibodies target cardiolipin, whereas anti-β_2_-glycoprotein 1 antibodies are directed against β_2_-glycoprotein 1, which acts as a cardiolipin-binding factor. LA is a mixture of autoantibodies that lead to the prolongation of phospholipid-dependent coagulation tests. APS is classified as either primary, when there is no evidence of associated diseases, or secondary, when it is associated with another systemic autoimmune disease, more commonly SLE. Thrombosis can affect any vascular bed, of all sizes, leading to arterial, venous, and microvascular thrombosis [7]. The most prevalent sites of venous and arterial thrombosis are the deep veins of the lower extremities and the cerebral circulation, respectively [8]; however, APS-related thrombosis can also occur in unusual sites, including visceral arteries and veins, as well as the cerebral venous sinuses. Obstetric APS increases the risks of adverse maternal and fetal outcomes and constitutes the most identified cause of recurrent pregnancy loss (RPL). It is also associated with premature birth, intrauterine fetal growth restriction, and maternal complications such as eclampsia or preeclampsia [9].

## 2. Pathophysiology

**Figure 1:**
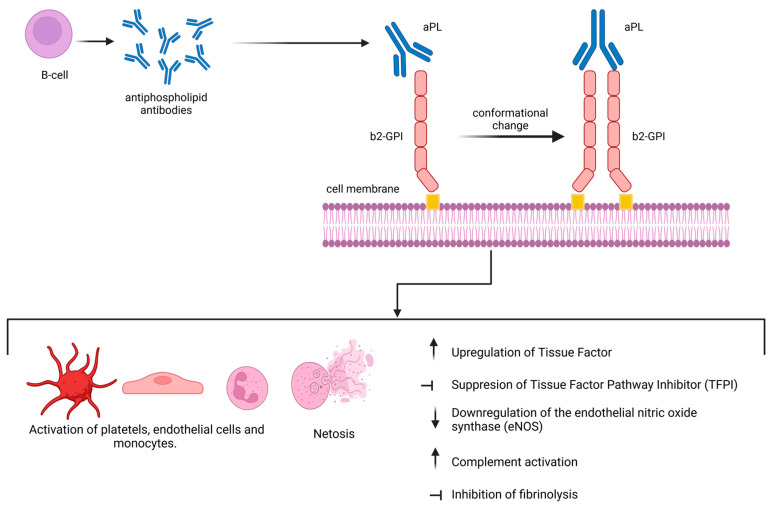
Binding of antiphospholipid antibodies to β2-GPI is a critical event in the pathogenesis of antiphospholipid syndrome. This interaction triggers a cascade of cellular activations, and initiates a wide array of responses, resulting in inflammation and thrombosis.

The pathophysiology of APS involves complex interactions among immune cells, endothelial cells, and the coagulation cascade (Figure 1). Antiphospholipid antibodies (aPLs) are produced by B-cells. Initially, it was believed that aPLs were able to bind anionic phospholipids such as cardiolipin and phosphatidylserine; however, it was later discovered that aPLs are directed against particular phospholipid-binding proteins such as β2 glycoprotein Ι (β2-GPI), prothrombin, annexin, etc. [10]. These antibodies interact with these proteins in a way that can activate endothelial cells, upregulate pro-coagulant factors, and stimulate inflammatory processes, significantly contributing to the pathogenesis of thrombosis seen in antiphospholipid syndrome [10,11].

In the realm of pathophysiology, the major target of aPL is epitopes expressed on β2GPI—also known as apolipoprotein H—a 326-amino-acid plasma protein produced mainly in the liver, which exhibits strong affinity for phospholipid surfaces, particularly when it forms dimers via reaction with an anti-β2 GPI antibody [12,13]. The β2GP1 consists of five domains (DI-DV). The fifth domain has a positive zone consisting of 14 lysines that specifically act as an anchor for anionic phospholipids [14]. Upon binding to anionic surfaces, the protein undergoes a conformational change to an open variation known as the J form, which is highly immunogenic. In this conformation, the DI epitopes are exposed to the environment, enabling the binding of antibodies [15]. Data available from several studies have demonstrated that aPLs activate various cell types such as endothelial cells, platelets, and monocytes, as well as inflammatory molecules [16]. In addition, various studies demonstrated that aPL can inhibit fibrinolysis and reduce activated protein C activity [17,18]. Binding of aPL to b2GPI on cellular surfaces leads to upregulation of genes encoding tissue factor, a cell surface glycoprotein that occupies a central position in hemostasis by initiating the extrinsic pathway of the blood coagulation cascade [19]. Moreover, binding of aPL in endothelial cells results in a cascade of events, including overexpression of adhesion molecules like E-selectin, downregulation of vasoprotective endothelial nitric oxide synthase (eNOS), and suppression of tissue factor pathway inhibitor (TFPI) activity [20,21].

Furthermore, aPLs are able to activate neutrophils, and the subsequent release of neutrophil extracellular traps (NETs), which correspond to neutrophil cellular death, has been acknowledged as a significant mediator of thrombosis. A notable finding was that monoclonal anti-β2-GPI antibodies effectively stimulated the production of NETs in vitro [22]. Finally, aPL can form immune complexes with phospholipid-binding proteins, activating the classical complement pathway, which enhances inflammation, hence contributing to the thrombogenic milieu in APS. Furthermore, complement factors might exacerbate thrombosis by facilitating endothelial activation and platelet aggregation, establishing a mechanistic connection to the thrombotic complications typical of APS [19].

Within the laboratory setting, aPL can be classified into two main groups: antibodies that are identified using solid-phase assays like aCL antibodies or anti-β2GPI antibodies, and antibodies that are identified based on their capacity to extend phospholipid-dependent coagulation tests, referred to as lupus anticoagulant (LA) [11]. Notwithstanding the fact that both in vitro and in vivo studies have shown evidence of the thrombogenic activity of aPL, their presence is not always associated with thrombotic events. Young, apparently healthy control subjects are reported to have a prevalence of 1 to 5 percent of aPL antibodies [12].

Moreover, several studies showed that mere presence or transfer of aPL is insufficient to induce thrombosis in experimental animals, suggesting that aPLs are necessary but not sufficient for the development of APS [23,24]. These observations made clinicians draw the conclusion that pathogenesis of APS follows a two-hit model. The “two-hit hypothesis” in antiphospholipid syndrome (APS) proposes that the development of APS requires two independent events: the first hit represents the persistently presence of aPL inducing a thrombophilic state whereas the second hit represents a stress condition, such as trauma, surgery, or infection, which pushes the hemostatic balance in favor of thrombosis [11].

Administration of pure anti-PL antibodies modifies the expression of endothelial adhesion molecules and disrupts vascular function, which is linked to TLR 2 and TLR4 signaling, as well as the increase of nitric oxide and tissue factor production. Consequently, infection or inflammation activate TLR signaling, and may elevate the expression of aPL epitopes, leading to an augmented risk for a clinical event. In obstetric APS, pregnancy per se could serve as the second hit [23,24]. It is important to remember that the two-hit hypothesis is a model, not a rigid rule. The exact interplay between genetic factors, environmental triggers, and immune responses in APS is still being investigated, and there is likely a degree of complexity beyond this simple model. The relative importance of each hit can also vary considerably between individuals.

## 3. Epidemiology

In light of the fact that the APS classification criteria have been subject to alterations, the lack of standardization to detect aPL, and other challenges, such as verifying aPL positivity twelve weeks after the original test, it has been difficult to estimate the exact frequency of APS. According to population studies, the estimated incidence and prevalence ranged between 1 and 2 cases per 100,000 and 40 and 50 cases per 100,000, respectively [25]. The prevalence of antiphospholipid antibodies was 10 percent in patients with deep vein thrombosis (DVT) and 14 percent in patients with stroke, while in cases of obstetric morbidity, it ranged from 6 to 9 percent [26].

Approximately 50 percent of individuals diagnosed with antiphospholipid syndrome (APS) have “primary APS”, while the remaining 50 percent of patients have “secondary APS” due to a concomitant systemic autoimmune illness—more commonly as a consequence of SLE [27]. The cardinal clinical manifestations of APS, including arterial or venous thrombosis and pregnancy complications, exhibit minimal variation overall, irrespective of whether the illness is primary or due to an underlying connective tissue disorder [28].

## 4. Clinical Manifestations

**Figure 2:**
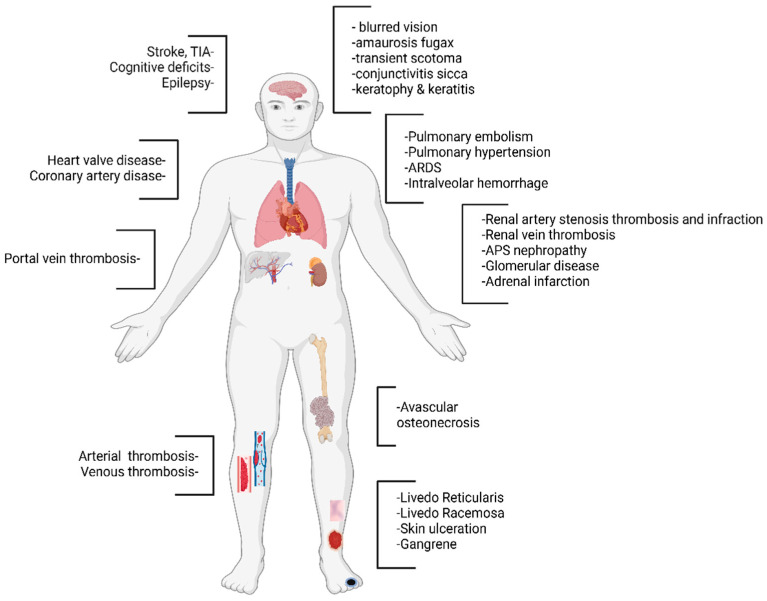
Clinical manifestations of APS.

Given the vascular nature of APS, the clinical presentations of the disease are rather diverse and can affect several organ systems (Figure 2).

### 4.1. Arterial and Venous Thromboembolic Disease

Thrombotic events are the hallmark of APS. In contrast to hereditary thrombophilias, which are most commonly associated with venous thromboembolism, APS can promote thrombosis in any vascular beds of all sizes, including arterial, venous, and microvascular circuits. Venous thrombosis, or embolism, is the most frequent manifestation. Large cohort studies have estimated that the deep veins of the lower limbs are the most prevalent sites of thrombosis, affecting between 20 and 30 percent of people with APS [8]. On the other hand, the most prevalent APS-related clinical manifestations in the arterial circulation are ischemic stroke and transient ischemic attacks (TIAs) [29,30]. It should be noted that APS accounts for up to 20 percent of stroke events in people under 45 years of age [31]. Thus, a thrombotic stroke occurring in a young patient without an obvious risk factor for cerebrovascular disease necessitates prompt investigation for APS. Interestingly, APS may also manifest with unusual site thrombosis, such as thrombosis in portal, renal, and mesenteric veins as well as cerebral venous sinus thrombosis [32].

Moreover, mesenteric artery occlusion, adrenal infarction, and avascular osteonecrosis have also been reported [33,34,35]. Of note, patients with APS can experience recurrent thrombotic events even despite therapeutic anticoagulation [36].

### 4.2. Thrombocytopenia

Thrombocytopenia is one of the most common hematological manifestations of APS, with an incidence ranging from 22 to 42 percent [37]. The consumption and/or destruction of platelets in the peripheral bloodstream, which occurs after the activation of platelets by APL, is assumed to be the cause of low platelet count in APS, which is usually mild and rarely requires treatment [38]. Of note, thrombocytopenia is likely associated with more severe disease, and prolonged mild to moderate thrombocytopenia is linked to decreased long-term survival [39,40].

### 4.3. Neurologic Manifestations

The nervous system is a major target of APS, and various neurologic manifestations have been linked with the disease that can arise either due to ischemia or thrombosis or as a consequence of direct injury to neurons by aPL. Cerebrovascular disease represents the most common cause of neurologic manifestations in APS and encompasses acute ischemic stroke, transient ischemic attack (TIA), and cerebral venous thrombosis (CVT) [41]. Stroke and TIA are the most common and severe arterial manifestations of APS, with cumulative prevalence of 19.8 and 11.1 percent, respectively [42]. According to a systematic review, the presence of aPL increased the risk for cerebrovascular events by 5.48-fold (95% CI 4.42 to 6.79) in patients less than 50 years old, making clear that in young patients who do not have any additional risk factors for atherosclerosis, the presence of TIA or ischemic strokes should raise suspicions for APS [43]. The primary causes of stroke and TIA in APS are mostly ascribed to either in situ thrombosis or cardioembolic processes due to valvular heart disease by deposition of immune complexes. When a cardiogenic source of emboli is not present, in situ thrombosis seems to be the most likely mechanism responsible for arterial blockage. Additionally, a pattern that is similar to vasculitis with multiple sites of narrowing and dilation has been described, which suggests that a concurrent vasculopathic process may be present in some situations [44]. CVT is a rare form of cerebrovascular event associated with APS, with a prevalence of 0.7 percent. However, 6–17 percent of CVT cases arise in the clinical setting of APS [45]. Other neurologic manifestations of APS include Sneddon syndrome, cognitive deficits, multiple sclerosis-like disease, Guillain–Barré syndrome, transverse myelopathy and myelitis, epilepsy, psychosis, and chorea [41,46,47].

### 4.4. Ocular Manifestations

Ocular manifestations are thought to be rare; however, in recent studies, it has been estimated to occur in up to 37 percent of affected individuals. Clinical findings vary depending on the affected part of the eye and include a wide spectrum of clinical manifestations from the anterior segment, such as conjunctivitis sicca, conjunctival vascular telangiectasias and microaneurysms, punctate epithelial keratopathy, and limbal keratitis, as well as blurred vision, amaurosis fugax, and transient scotoma when the posterior segment of the eye is involved [48,49].

### 4.5. Cutaneous Manifestations

A variety of cutaneous findings have been described in association with APS, and in some cases may be the initial presentation of APS. Among other skin conditions, livedo reticularis, a purple reticular rash that is more frequently identified on the limbs, trunk, and buttocks, is the most common skin manifestation [50]. Livedo reticularis arises as a result of impaired blood flow in cutaneous vessels and has been associated with multiple thromboses and stroke [51]. It has a prevalence of 25 percent in patients with primary APS and 70 percent in patients with SLE-associated APS [50]. Other cutaneous manifestations include livedo racemosa (coarser and more irregular pattern of purple reticular rash), digital gangrene, splinter hemorrhages, cutaneous necrosis, ulceration, and pyoderma gangrenosum-like skin lesions [52].

### 4.6. Cardiac Manifestations

Cardiac manifestations of APS most commonly involve the valves, including valvular thickening and valvular nodules or nonbacterial vegetations (Libman–Sacks endocarditis), principally affecting the mitral valve, with the aortic valve following closely behind [53,54]. Heart valve disease in the setting of APS—especially aortic nodules—is related to an increased risk of stroke [55]. Furthermore, apart from the widely noted valve abnormalities, individuals with APS exhibit a higher prevalence of ischemic heart disease compared to the general population. Myocardial infarction can arise as a result of coronary thrombosis in the absence of underlying atherosclerosis, accelerated atherosclerosis leading to atherothrombosis, or microvascular injury. In the APS registries, myocardial infarction is observed in approximately 5.5 percent of individuals involved [27,56].

### 4.7. Pulmonary Manifestations

Several pulmonary manifestations can develop in patients with APS as a result of vascular or parenchymal involvement. Pulmonary embolism (PE) is the predominant pulmonary manifestation, affecting up to 14 percent of APS patients [27]. Other less common pulmonary manifestations include pulmonary hypertension, acute respiratory distress syndrome (ARDS), and intra-alveolar hemorrhage [57,58,59,60].

### 4.8. Renal Manifestations

APS can be associated with a broad spectrum of renal manifestations, including hypertension, large vessel disease (renal artery stenosis, thrombosis, and infarction; renal vein thrombosis), intrarenal vascular lesions (APS nephropathy), and glomerular disease. Of note, the exact incidence of renal involvement varies due to the constraints of histopathological studies, contraindications for biopsy, and its correlation with SLE [60,61].

### 4.9. Catastrophic APS

Catastrophic APS (CAPS), also known as Asherson’s syndrome, represents the most severe form of APS, affecting approximately less than 1 percent of patients and can be the first manifestation of APS. CAPS represents a medical emergency, associated with a high mortality rate of up to 50% [27,60]. The cardinal feature of CAPS is the rapid onset of devastating, diffuse, thrombotic sequelae affecting both the venous and arterial systems in a micro- or combined micro- and macrovascular process over a short period of time. This “thrombotic storm” results in extensive tissue damage and multi-organ failure. CAPS is defined by clinical involvement of at least three different organ systems with histologic evidence of thrombosis, with more common sites of involvement including the kidney, lung, central nervous system, heart, and skin. Most of our understanding about CAPS derives from the CAPS Registry, an online database established to enhance awareness of this entity. According to the registry, in nearly half of these cases, CAPS was the initial presentation of APS [62]. Approximately one-half of all patients with CAPS have a prior diagnosis of APS, and 30 percent were known to have had a prior diagnosis of SLE. In general, females are more susceptible than males, comprising roughly 70 percent of cases compared to 30 percent for males [62,63]. Precipitating factors for the development of CAPS encompass infections, surgery/trauma, malignancies, pregnancy, estrogen use, and subtherapeutic anticoagulation in APS patients [64]. Mutations in complement regulatory genes have been identified as a potential risk factor for the development of CAPS, as it was further substantiated by reports indicating successful outcomes with eculizumab in a subset of patients who are refractory to standard treatment [65].

Differential diagnosis of CAPS includes other thrombotic syndromes, notably those leading to venous, arterial, and microvascular thrombosis in multiple or uncommon sites, such as disseminated intravascular coagulation (DIC), heparin-induced thrombocytopenia (HIT), thrombotic microangiopathies (TMA), sepsis, and HELLP (hemolysis, elevated liver enzymes, and low platelets) syndrome [64,66,67].

### 4.10. Obstetric APS

Nilson et al. first documented the correlation between recurrent spontaneous abortions and LA almost 50 years ago [68], while Graham Hughes first established the relationship between aCL antibodies and miscarriages in 1984 [69]. It is now well established that obstetric APS (OAPS) is the most identified cause of recurrent pregnancy loss and late-pregnancy morbidity related to placental injury. The APS ACTION group demonstrated that 6% of patients experiencing relevant pregnancy morbidity tested positive for antiphospholipid antibodies [70]. The presence of antiphospholipid antibodies is linked to obstetric morbidity such as intrauterine growth restriction, preeclampsia, chorea gravidarum, and recurrent spontaneous fetal loss. The pathogenesis of pregnancy morbidity is incompletely understood, but inflammation, complement activation, and placental thrombosis have all been suggested to contribute to the pathogenesis of OAPS [71].

## 5. How to Set the Diagnosis

To diagnose or classify an individual with APS, clinicians have traditionally utilized the Sapporo criteria, which highlight both clinical and laboratory components. The Sapporo criteria were originally established in 1999 and were subsequently revised at the Sydney International Antiphospholipid Antibodies Congress in 2006 and are referred to as the updated Sapporo or Sydney criteria (Table 1) [72,73].

However, these criteria exhibit shortcomings in that, for example, they do not adequately recognize the difference between IgG and IgM (IgG autoantibodies are better associated with thrombotic APS whereas IgM with obstetric APS), do not emphasize late- versus early-pregnancy morbidities, and lack recognition of small–microvascular clotting manifestations, along with other laboratory or imaging clues for APS.

In 2023, the American College of Rheumatology (ACR) and the European Alliance of Associations for Rheumatology (EULAR) established new classification criteria for APS [74]. The new criteria aim to assess the significance of the various clinical and laboratory elements, as well as taking into account patients’ history. For instance, if an individual experiences a venous thromboembolism (VTE) following hip replacement surgery, this evidence is not as compelling for a diagnosis of antiphospholipid syndrome (APS) compared to a case where a VTE occurs without provocation or in the absence of a transient major risk factor. Moreover, additionally acknowledge points for biopsy-confirmed microvascular involvement without alternative explanations. Furthermore, the revised criteria place significant emphasis on numerous points of clinical certainty regarding late pregnancy complications, while giving considerably less attention to early fetal losses. Cardiac valve abnormalities and thrombocytopenia serve as indicators included in the criteria to assist clinicians in identifying this syndrome. Rare but important clinical phenomena are also included, e.g., diffuse alveolar hemorrhage, which is a rare but well-described complication of APS. Finally, there is a notable de-emphasis on cases of IgM-only positivity [74].

The ACR/EULAR classification criteria include an entry criterion of at least one positive antiphospholipid antibody test within 3 years of an APS-associated clinical criterion. These criteria are supplemented by additional weighted criteria, each scoring between 1 and 7 points, organized into six clinical domains, macrovascular venous thromboembolism, macrovascular arterial thrombosis, microvascular, obstetric, cardiac valve, and hematologic, along with two laboratory domains: lupus anticoagulant tests and IgG/IgM anticardiolipin or IgG/IgM β2GP1b. Patients with APS are those who score at least 3 points in both clinical and laboratory domains.

The newly established criteria aim to improve diagnostic accuracy and patient outcomes through a more systematic and evidence-based approach [74,75], primarily intended for patient classification in research studies. The other goal is to recognize that there are different subtypes of APS, and this also may have important implications for research. It might be that our therapeutic approach to obstetric APS should be different from our approach to thrombotic APS and vice versa. In real-world data, the 2023 ACR/EULAR APS classification criteria demonstrate high specificity but low sensitivity when compared to the revised Sapporo criteria. This difference is attributed to stricter criteria for thrombosis risk assessment, obstetric events, and laboratory findings. Evidence suggests that the ACR/EULAR criteria are more suitable for research due to their high specificity in defining homogenous APS cohorts, whereas the Sapporo criteria remain valuable for clinical diagnosis given their higher sensitivity [75].

We believe that while they may not serve as the definitive factor in clinical decision-making, they provide an additional tool to assist clinicians in determining whether to consider or exclude APS in specific patient cases. For this reason, clinicians are encouraged to familiarize themselves with these criteria, whereas regular updates and ongoing feedback can help refine these over time.

In daily clinical practice, clinical scenarios that might heighten clinical suspicion for APS, requiring prompt investigation, are thrombotic events in the venous, arterial, or microvascular system, otherwise unexplained, especially in young adults (<50 years old) and in individuals with autoimmune diseases; thrombotic events in unusual sites; and recurrent miscarriage.

In patients with suspected antiphospholipid syndrome (APS), it is imperative to obtain a comprehensive medical history and physical examination and to perform laboratory assessment for the detection of aPL. Although there are no pathognomonic physical symptoms of APS, the examination may disclose findings associated with ischemia or infarction of particular organs, such as the skin (e.g., livedo reticularis).

The presence of aPL in the appropriate clinical setting (venous thrombosis, arterial thrombosis, or pregnancy morbidity) is the hallmark laboratory test result that establishes the diagnosis of APS [72].

Antibody testing in patients with suspected APS entails two immunoassays and a functional coagulation assay: IgG or IgM anticardiolipin antibodies (detected by ELISA), IgG or IgM anti-β2-glycoprotein 1 antibodies (detected by ELISA), and a lupus anticoagulant functional coagulation assay [76]. LA assays detect antiphospholipid antibodies that interfere with and prolong clotting times with phospholipid-dependent clotting tests such as dRVVT and APTT [77].

LA presence is confirmed if the prolonged aPTT or dRVVT persists even after mixing the patient’s plasma with normal platelet-poor plasma or if it is significantly shorter after the addition of phospholipid [78]. LA and high-titer aCL antibodies have similar clinical implications, although studies suggest a higher thrombotic risk in patients with LA. Antibodies or LA functional coagulation assay must be detected in subsequent testing conducted at least 12 weeks apart, as they may transiently manifest in other situations (e.g., infections) [29].

## 6. Management

APS requires a multidisciplinary approach due to its diverse clinical manifestations and potential for severe complications [79]. Effective management necessitates collaboration among several specialists, including hematologists, rheumatologists, obstetricians/gynecologists, neurologists, and nephrologists in a patient-centered manner. The multidisciplinary approach in practice involves regular monitoring and risk stratification in order to assess individual risk for thrombosis (primary and secondary prevention) and other complications for treatment optimization as well as to tailor treatment intensity. Of note, involved physicians have to provide clear information about APS and its potential complications and the importance of adherence to treatment plans. This collaborative approach ensures comprehensive care, minimizes complications, and improves the quality of life for individuals with APS.

### 6.1. Primary Prevention

The presence of aPL is not exclusive to APS, and interestingly, the prevalence of aPL antibodies in the general population is approximately 1–5% [12]. Moreover, LA and aCL antibodies have been reported in a variety of clinical disorders. In daily clinical practice, the issue of primary thrombosis prevention arises when a patient tests positive for aPL for reasons unrelated to an acute thrombosis evaluation such as SLE or unexplained prolongation of aPTT. In such cases, it is important to define which aPL is present (single or multiple) as well as the titer of the autoantibodies. Another important aspect is the persistence of aPL positivity in repeated measurements [79]. All the above parameters constitute the so-called individual aPL profile, which represents an important factor for risk stratification for thrombotic and obstetric events. A high-risk aPL profile is defined by the presence of persistent positivity for LA, double (any combination of LA, aCL, or anti-β2 GPI antibodies) or triple (i.e., all three subtypes) positivity, or persistently high aPL titers [80]. According to the EULAR recommendations, prophylactic therapy with low-dose aspirin (75–100 mg daily) is recommended in individuals with a high-risk aPL profile regardless of conventional cardiovascular risk factors [79], as demonstrated by a meta-analysis of seven observational studies of 460 asymptomatic aPL carriers where low-dose aspirin administration was associated with a 50% decrease in the incidence of first thrombosis [81]. Low-dose aspirin administration is also recommended in individuals with a history of SLE with a high-risk aPL profile and a non-known history of thrombosis or pregnancy complications, whereas it could be an option in the absence of a high-risk aPL profile. Finally, in non-pregnant women with a history of obstetric APS (with or without SLE), prophylactic therapy with low-dose aspirin is recommended following a sufficient risk/benefit assessment [79].

### 6.2. Secondary Prevention

In patients with APS and VTE, following initial treatment with unfractionated or low molecular weight heparin (LMWH) and a bridging therapy involving heparin plus vitamin K antagonists (VKAs), current guidelines recommend long-term administration of vitamin K antagonists (VKAs) with a target INR of 2.0 to 3.0 [82]. In cases of VTE during pregnancy, LMWH at therapeutic doses, adjusted periodically based on body weight, is preferred over VKAs, which are contraindicated due to their embryotoxic effects. Long-term administration of VKAs with a target INR of 2.0 to 3.0 should also be given in cases of arterial thrombosis outside the cerebral circulation, whereas secondary prevention for stroke patients could be tailored to their individual risk profiles and can be achieved through either administration of low-dose aspirin or VKAs or, in some instances, both [83].

Although the use of DOACs for secondary thrombosis prevention has become more prevalent in the general population, there is insufficient evidence regarding their use in patients with APS. In a post-hoc analysis of patients with APS from three randomized controlled trials (RCTs) comparing dabigatran to warfarin, along with one trial evaluating rivaroxaban against warfarin in venous thrombotic APS, there was no indication of significant differences in outcomes between direct oral anticoagulants (DOACs) and VKAs for venous thrombosis [84,85,86]. However, the reliability of these findings is constrained by small sample sizes, insufficient representation of high-risk APS patients, and brief follow-up durations [74]. Moreover, in an RCT comparing rivaroxaban with warfarin in patients with triple aPL, positivity was prematurely halted due to a higher incidence of thromboembolic events, primarily arterial, in the rivaroxaban group [74,83].

The duration of anticoagulation treatment depends on whether the thrombotic event is provoked or unprovoked. Individuals with APS and an unprovoked thrombotic event should be recommended lifelong anticoagulation, whereas when there is an identifiable factor, treatment should be maintained for a duration advised for individuals without APS [79].

APS patients can experience recurrent thrombosis even when receiving antithrombotic therapy. However, there is a lack of high-quality data to endorse a specific therapeutic approach in this scenario. Possible alternative approaches could include escalating VKA therapy to a target INR of 3–4, incorporating aspirin, employing an alternative anticoagulant such as low molecular weight heparin, or introducing other agents such as statins due to their pleiotropic effect or hydroxychloroquine [87].

## 7. Conclusions

APS is a thrombo-inflammatory autoimmune disorder characterized by autoantibodies that target cell surface phospholipids and phospholipid-binding proteins and is associated with a wide range of thrombotic and non-thrombotic manifestations as well as obstetric complications. APS classification criteria were developed for research purposes; however, they can assist diagnosis, which is based on clinical and laboratory findings in the appropriate setting. VKAs continue to be the cornerstone of management for the vast majority of patients with APS, and according to current evidence, seem to surpass direct oral anticoagulants. Advancements in understanding the mechanisms by which autoantibodies induce disease have led to the identification of potential therapeutic targets within the innate immune system, specifically focusing on the complement system and neutrophil extracellular traps (NETs).

## Figures and Tables

**Table 1:** The 2006 revised Sapporo criteria for APS.

2006 Revised Sapporo Criteria [73]: At least 1 clinical criterion and 1 laboratory Criterion
Clinical criteria
1. Vascular thrombosis: One or more clinical episodes of arterial, venous, or small vessel thrombosis in any tissue or organ. 2. Pregnancy morbidity One or more unexplained deaths of a morphologically normal fetus at or beyond the 10th week of gestation, with normal fetus morphology documented by ultrasound or by direct examination of the fetus. One or more premature births of a morphologically normal neonate before the 34th week of gestation because of (i) eclampsia or severe preeclampsia defined according to standard definitions, (ii) recognized features of placental insufficiency, or (iii) recognized features of placental insufficiency. Three or more unexplained consecutive spontaneous abortions before the 10th week of gestation, without maternal or hormonal abnormalities, and paternal and maternal chromosomal causes excluded.
Laboratory criteria
LA present in plasma, on 2 or more occasions at least 12 weeks apart aCL of IgG and/or IgM isotype in serum or plasma, present in medium or high titer, on 2 or more occasions, at least 12 weeks apart, measured by a standardized enzyme-linked immunosorbent assay (ELISA). Anti-beta2 glycoprotein I antibody of IgG and/or IgM isotype in serum or plasma (in titer >the 99th percentile), present on 2 or more occasions, at least 12 weeks apart, measured by a standardized enzyme-linked immunosorbent assay (ELISA)
